# Deriving the A/B Cells Policy as a Robust Multi-Object Cell Pipeline for Time-Lapse Microscopy

**DOI:** 10.3390/ijms26178455

**Published:** 2025-08-30

**Authors:** Ilya Larin, Egor Panferov, Maria Dodina, Diana Shaykhutdinova, Sofia Larina, Ekaterina Minskaia, Alexander Karabelsky

**Affiliations:** 1 Translational Medicine Research Center, Sirius University of Science and Technology, Federal Territory Sirius, Olympic Ave. 1, 354340 Sirius, Russia; 2 IT-College, Sirius University of Science and Technology, Federal Territory Sirius, Olympic Ave. 1, 354340 Sirius, Russia

**Keywords:** MSC, A/B models, descriptive statistics, cell tracking

## Abstract

Time-lapse microscopy of mesenchymal stem cell (MSC) cultures allows for the quantitative observation of their self-renewal, proliferation, and differentiation. However, the rigorous comparison of two conditions, baseline (A) versus perturbation (B) (the addition of molecular factors, environmental shifts, genetic modification, etc.), remains difficult because morphology, division timing, and migratory behavior are highly heterogeneous at the single-cell scale. MSCs can be used as an in vitro model to study cell morphology and kinetics in order to assess the effect of, for example, gene therapy and prime editing in the near future. By combining static, frame-wise morphology with dynamic descriptors, we can obtain weight profiles that highlight which morphological and behavioral dimensions drive divergence. In this study, we present A/B Cells Policy: a modular, open-source Python package implementing a robust cell tracking pipeline. It integrates a YOLO-based architecture as a two-stage assignment framework with fallback and recovery passes, re-identification of lost tracks, and lineage reconstruction. The framework links descriptive statistics to a transferable system, opening up avenues for regenerative medicine, pharmacology, and early translational pipelines. It does this by providing an interpretable, measurement-based bridge between in vitro imaging and in silico intervention strategy planning.

## 1. Introduction

Brightfield microscopy is one of the simplest and least invasive methods of live cell imaging, yet it is often overlooked in favor of fluorescence-based techniques. Relying solely on transmitted light, brightfield illumination captures cell morphology, boundary contours, and gross refractive index variations, eliminating the need for exogenous labels or dyes. This label-free approach enables the continuous, long-term observation of live cells with minimal phototoxicity or disturbance to normal physiology. In time-lapse experiments, brightfield images provide a robust basis for automated segmentation and centroid tracking, yielding reliable measurements of area, shape, and displacement that can be compared directly across experimental conditions.

Phase-contrast microscopy builds on this by converting the minute phase shifts generated as light passes through transparent cellular structures into intensity differences, providing enhanced contrast. Subcellular features such as lamellipodial protrusions, stress fibers, and intracellular granularity become visible even in unstained samples. This enables the extraction of dynamic morphological features (e.g., eccentricity fluctuations and orientation changes) that would otherwise require complex staining protocols. Its ability to reveal both the static and dynamic aspects of cell behavior combined with a high temporal resolution, gentle illumination, and rich morphological content, makes phase-contrast microscopy an ideal tool for quantifying migration kinetics, division timing, and differentiation events in MSC cultures, particularly when paired with advanced tracking pipelines such as comparing baseline (A) versus perturbations (B) [1].

In frame-by-frame imaging and cell analysis, static features describe cell properties at a given point in time, while dynamic features characterize how these properties evolve or how the cell moves over time. Static descriptors answer the question, ‘What is this cell at the moment?’ They capture morphology or state classification models (e.g., cell shape, size, or phenotype in a single frame). In contrast, dynamic metrics answer the question ‘How is it changing or moving?’ They capture cell behavior over time or process models (e.g., movement paths, velocity, turning angles, and fluctuations).

Brightfield live cell imaging produces long, label-free sequences in which each frame constitutes a sample and the time axis encodes latent progression. The quantitative analysis of cell behavior from time-lapse microscopy typically begins with descriptive statistics: per-frame (static) morphology and derived trajectory (dynamic) features such as area, circularity, elongation, velocity, directional persistence, division events, etc. Traditional population-level summaries (mean size, average speed, and cumulative division count) conceal substantial intra-population variability and obscure transient or branching phenotypic paths. High-content profiling has expanded from handcrafted metrics to rich neural embeddings integrating fluorescence or brightfield cues with textual or contextual annotations [2]. Manifold and archetype-based representations in non-Euclidean spaces improve invariance and equivariance handling for cell grouping [3]. Nevertheless, classical reaction–diffusion or growth curve models describe aggregate density or area but do not reveal the latent decision structure by which individual cells switch between spreading, migration, division, quiescence, or differentiation. Such pipelines can be especially useful for analyzing cells that display complex phenotypic variations in response to even minor changes in culture conditions, such as MSCs [4]. MSC identity is defined by a set of expressed surface markers, plastic adherence, and multipotent differentiation potential [5] and exhibits donor- and tissue-of-origin heterogeneity [6,7,8,9]. MSCs from different sources differ in morphology, proliferation kinetics, the timing of senescence onset, and secretome composition, complicating direct cross-condition comparisons if only simple averages are used. This approach is especially valuable for diseases for which modeling may also require the assessment of cell differentiation. Such a strategy may sometimes be the only means of evaluating the therapy efficiency by using indirect metrics, whether static or dynamic. In this study, we focused on a SOTA bridge between descriptive and time-dependent statistics. The statistical layer retains rigor: frame-wise distributional analyses (including multimodality, tail behavior, and outlier persistence), sliding-window smoothing with effective sample size corrections for autocorrelation, non-parametric blocked tests (Friedman’s with appropriate post hoc adjustment), and distributional distances (Kolmogorov–Smirnov’s and Wasserstein’s) provide a calibrated detection of divergence intervals. These intervals inform where successor weights should be inspected for shifts, guiding active experimental design decisions such as adjusting frame rate for phases with rapid successor change or allocating annotation effort to cells occupying high forward–backward uncertainty regions. In this framework, the addition of a perturbation modifies the weighting over shape dynamics (the increased importance of spreading kinetics or orientation alignment) while preserving base occupancy trajectories; thus, extrapolation and counterfactual simulation remain stable.

## 2. Results and Discussion

### 2.1. A/B CellTracker

A/B testing originated in marketing and IT development as a means of evaluating the impact of changes by randomly allocating users to different groups and comparing key metrics. The classic approach assumes that each observation unit is independent and the response to the change can be described by a simple additive model. However, these assumptions are often violated in many applied fields, ranging from medicine to cell biology, due to the complex nature of the data, nonlinear effects, and temporal dependencies.

In biological research, significant heterogeneity can be introduced by the experimental system itself: different cell types respond differently to stimuli; the population already consists of different subtypes; and microenvironmental conditions (cell density and local nutrition) can vary within a single Petri dish [10]. Blind A/B testing, in which cells or cultures are randomly distributed between control and experimental conditions without a prior analysis of the quality and distribution of metrics can lead to false conclusions. For example, this can occur when one experimental group has more of the highly migratory cells by chance, or when a long series of measurements over time creates strong autocorrelation and cross-correlation.

These issues become even more pronounced when analyzing time-lapse microscopy data, where the position, shape, and dynamic parameters of each cell are recorded as it is tracked across hundreds of frames. Simply aggregating all control frames with all experimental frames ignores the nested data structure (tracks → frames), temporal autocorrelation, and heterogeneity across cells and over time. In order to correctly assess the effect of exposure to a drug, for example, it is necessary to combine randomization methods to avoid systematic biases, using advanced statistical models (such as mixed effects models, nonlinear generalized models, or spline curves for temporal dynamics) and non-parametric approaches (Friedman’s permutation tests or bootstrapping within blocks). This comprehensive approach enables us to consider the hierarchy (cell → trajectory → series of frames), account for autocorrelation, and plan a sufficient sample size to detect small and large nonlinear effects in the temporal evolution of cell behavior.

The A/B CellTracker initializes a highly configurable cell tracking environment in which global constants govern everything from the minimum detectable object area to the maximum tolerated gap for re-identification. This ensures that the pipeline can be tuned for different imaging conditions and cell types.

For each frame, the code divides large images into overlapping tiles, applies a pre-trained YOLO model to segment candidate cell regions, and then stitches and cleans these polygons together through duplicate suppression and merge–overlap routines. For each valid polygon, the code computes a 24-dimension feature vector encompassing intensity statistics, Hu moments, normalized central moments, solidity, aspect ratio, and fill factor. This vector is then L2 normalized. These features, together with polygon intersection over union (IoU) and centroid positions, serve as inputs to a custom cost–matrix assignment process that balances appearance, shape, and spatial proximity. This process is augmented by adaptive gating and fallback strategies to handle ambiguous or low-confidence matches.

The core tracking loop maintains a list of active tracks, each of which is represented by a small stateful object that stores its polygon, exponentially weighted feature and area histories, velocity estimates, and generation index. At each time step, the algorithm uses the Hungarian method to compute an optimal assignment between existing tracks and new detections, applies multi-stage recovery passes to address splits, merges, and missed detections, and archives stale tracks for potential re-identification in future frames. Finalized track updates, including confirmed lineage splits, persist in the database alongside frame-level metrics (e.g., track count, average IoU, splits, merges, ID switch events, and continuity scores), and optional annotated images are saved to provide a complete audit trail for downstream quantitative analysis.

### 2.2. A/B CellAnalyser

The A/B CellAnalyser-segmented cell is initially linked to the exact moment it was observed. These time-stamped masks are then used to reconstruct the trajectories of individual cells across the image sequence. By aligning the contours of the cells to their respective time points, we can observe how their morphological and positional characteristics evolve, providing a detailed view of their shape and long-term motility behavior.

At each time point, we quantify the cell’s two-dimensional footprint by computing its projected area and boundary length, converting pixel measurements into micrometers. From these basic geometric measurements, we derive a dimensionless shape factor that reflects how closely the cell approximates a perfect circle. We then approximate the contour with an ellipse to extract its elongation (eccentricity) and its principal orientation relative to the image axes. After centering and variance scaling these descriptors, each cell in each frame is represented by a concise feature vector that highlights subtle differences in size, circularity, elongation, and tilt.

By tracking each cell over time, we can transform its sequence of centroids into a trajectory in physical space. The squared displacement of each position from its origin yields a mean squared displacement profile, while the total path length and net displacement provide an indication of the straightness of the path. Examining successive motion vectors enables us to compute instantaneous speeds and accelerations, detect sharp changes in direction that exceed a biologically meaningful threshold, and evaluate the tendency to maintain directionality by calculating the average cosine of turning angles. We identify periods of almost no movement by setting a low speed threshold, which characterizes pauses or arrests in motion.

Beyond these primary motility indices, we examine more complex dynamic signatures. The variability of speed over time, the fractal complexity of the cell’s wandering path, and the statistical coupling between shape fluctuations and speed changes reveal the interplay between morphological and kinematic processes. Cross-correlations between orthogonal velocity components further illuminate coordinated motion patterns, while the overall ratio of directional displacement to path length captures migration efficiency.

By merging these static and dynamic feature spaces, we obtain a rich, high-dimensional portrait of cell behavior. Such comprehensive phenotyping supports clustering and comparative analysis across experimental conditions and provides empirical inputs for mechanistic models—ordinary differential equations whose parameters can be adjusted to reproduce observed migration and morphology dynamics. The Hungarian algorithm and Kalman filters are being optimized currently so that they can be used to track each individual cell during brightfield and phase-contrast imaging. This integrative approach paves the way for a deeper quantitative understanding of how cells navigate, deform, and interact with their microenvironment over time.

### 2.3. A/B Cell Collection: Description and Metadata

MSCs are known to exhibit a significant degree of heterogeneity when exposed to different cell culture conditions. This is exacerbated further by the initial heterogeneity of primary MSCs; many studies show that even human MSC colonies derived from a single cell contain at least three distinct cell types: small, rapidly renewing cells; elongated, spindle-like cells; and large, slow-renewing cuboidal cells [11]. Furthermore, the subsequent expansion of these colonies results in a dramatic decrease in the diversity of observed cell types, eventually producing subpopulations that do not represent the most abundant clones observed at early passages [12].

The other important heterogeneity-inducing factor is the supplementation of the media. For example, fetal bovine serum (FBS), which is still the most commonly used cell culture supplement, owing to its rich supply of growth factors, cytokines, and chemokines, is known to possess a great deal of batch-to-batch variability, and the same is true for many of its replacements, such as human platelet lysate. As a result of these discrepancies in media composition, many properties of MSCs can be affected. Proliferation rate, the timing of senescence onset, morphology, gene expression patterns, and proliferation capacity have all been shown to be impacted by the exact composition of the supplement [13,14].

In our study, we compared mouse bone marrow MSCs (bmMSC) by changing three variables: passage (8 and 10), FBS concentration in the culture medium (5% and 10%), and cell seeding density (5000 and 10,000 cells per well of a 48-well plate). Although these factors are seemingly relatively minor, they introduced measurable morphometric differences between cells in different conditions, as evidenced by descriptive statistics and a simulation model. We attribute these differences to the varying concentrations of functional molecules due to the different FBS concentrations, as variations in these concentrations are known to affect the phenotype of MSCs [15]. We also attribute these differences to the spontaneous changes that occur with repetitive passaging of MSCs [16], a process that has previously been shown to be delayed by a low plating density [17].

**Figure 1:**
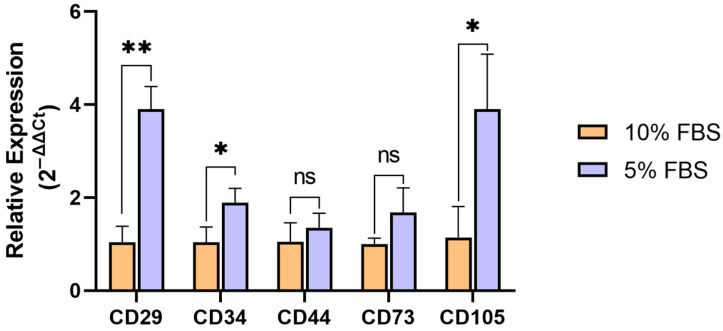
Relative expression of bmMSC markers in cells cultured in media with the addition of 5% or 10% FBS. *—*p* < 0.05, **—*p* < 0.01; ns—not significant.

To assess the impact of FBS concentration on the MSC phenotype, we analyzed the expression of key MSC markers using qRT-PCR. Passage 8 MSCs were cultured in media containing either 5% or 10% FBS. The following markers were analyzed: CD29, CD34, CD44, CD73, and CD105. According to the formal ISCT (International Society for Cellular Therapy) criteria [18], MSCs are expected to be positive for CD29, CD44, CD73, and CD105, and negative for CD34. The observed results (Figure 1) demonstrate that the reduction in FBS concentration leads to the elevated expression of CD29, CD34, and CD105. The elevation of the MSC-nonspecific CD34 marker may mark the partial loss of MSC identity, while elevated CD29 (integrin beta-1) expression seems to imply an increase in MSC adhesion to plastic, which is likely an adaptive reaction to a reduction in nutrient concentration. Fluctuations in CD105 expression level are known to occur in MSCs in response to different culture conditions [19]; however, their biological significance remains largely unclear.

**Figure 2:**
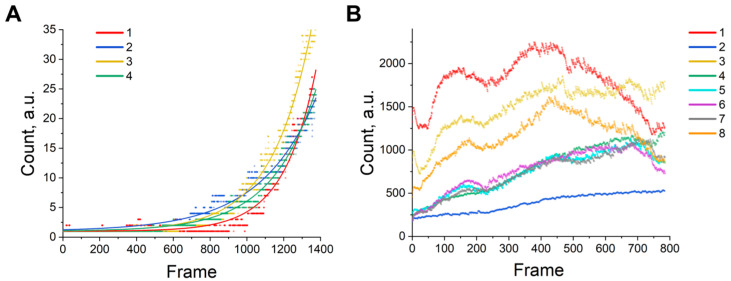
(**A**) The number of cells in the mmSC dataset (abbreviations: 1-01, 2-02, 3-test01, 4-test02), (**B**) the number of cells in the bmMSCs dataset (abbreviations: 1-P8, 5K, 5%FBS; 2-P8, 5K, 10%FBS; 3-P8, 10K, 5%FBS; 4-P8, 10K, 10%FBS; 5-P10, 5K, 5%FBS; 6-P10, 5K, 10%FBS; 7-P10, 10K, 5%FBS; 8-P10, 10K, 10%FBS).

MSCs in all four time-lapse experiments exhibited dynamic changes in morphology and behavior over the course of imaging. We first quantified basic shape descriptors—cell spread area, circularity, and orientation—to capture these changes. Despite similar initial conditions, clear differences emerged between experiments in both the average trends and distributional properties of these features. We are addressing a common issue: are there any differences between the series of experiments? Rather than making independent observations, we are working with a time series, where neighboring frames are clearly correlated. Furthermore, due to the delays between the imaging of individual wells of a culture plate introduced as a result of the cumulative time spent on the acquisition of images from the previous well, there is a phase lag and an in-series phase shift (the frames describe different physical moments in time). In N_eff_, 95% of windows are eliminated when *p* > α (Supplementary Files S1 and S2), and the permutation version of Friedman’s test provides control the first-kind error. We demonstrate the feasibility of this pipeline using the publicly available Cell Tracking Challenge dataset of Helen Blau’s team [20] generated from mouse muscle stem cells (mmSCs) in hydrogel microwells (Figure 2A), which was labeled by our team. In this example, experiments 1 and 2 were labeled. Data from experiments 3 (test01) and 4 (test02) were obtained using the YOLO11 convolutional architecture, which was trained using the labeled data from experiments 1 and 2. We also used mouse bmMSCs (Figure 2B): 1 (passage 8, 5K cells, 5% FBS), 2 (passage 8, 5K cells, 10% FBS), 3 (passage 8, 10K cells, 5% FBS), 4 (passage 8, 10K cells, 10% FBS), 5 (passage 10, 5K cells, 5% FBS), 6 (passage 10, 5K cells, 10% FBS), 7 (passage 10, 10K cells, 5% FBS), and 8 (passage 10, 10K cells, 10% FBS).

The differentiation frequency constant ranges from 0.0066 to 0.24 (per frame) for the experiments conducted by the team of Helen Blau. The differentiation frequency is a universal tool for interpreting various cell metrics. A typical growth curve includes a lag phase (adaptation and low growth), a logarithmic phase (exponential growth in number and total area occupied), and a plateau phase (stationary growth) once 100% coverage has been reached. In the case of mmSCs, it is convenient to use an exponential equation. The number of cells per frame grows steadily through the bmMSCs experiment, with most populations eventually becoming overconfluent to the point where cell clumping is followed by mass cell death.

**Figure 3:**
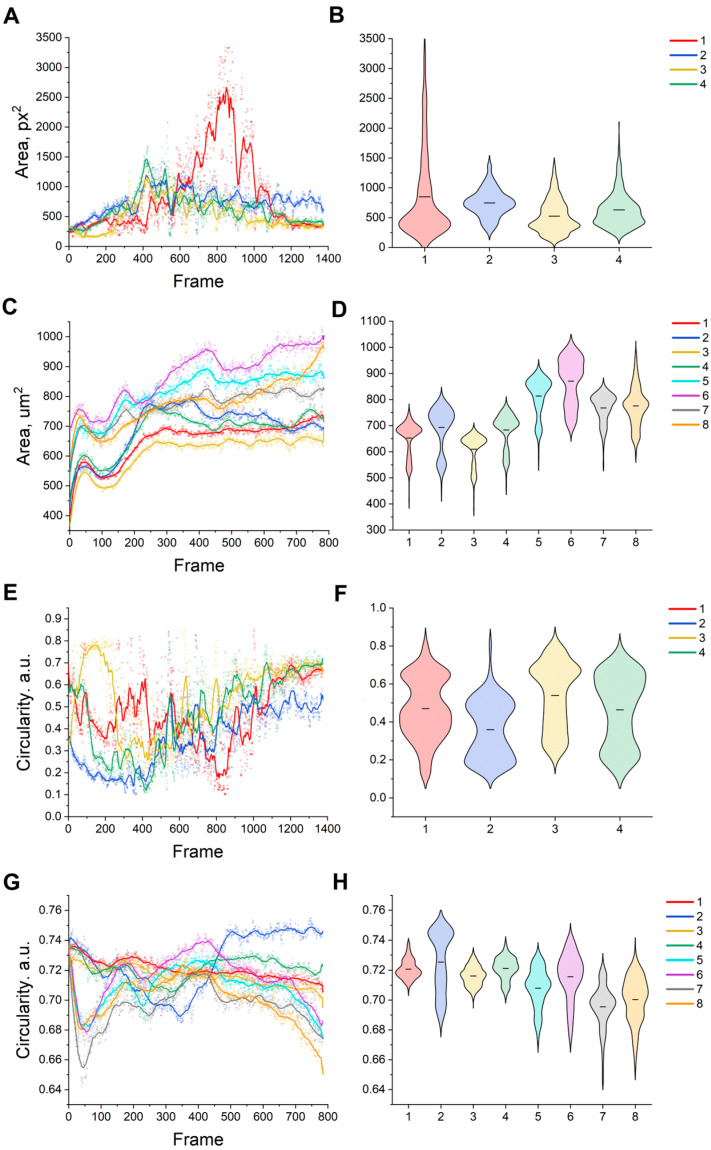
(**A**) The mmSC cell area over time. (**B**) The violin plot of the area for all frames within the mmSC series. (**C**) The bmMSC cell area over time. (**D**) The violin plot of the area for all frames within the bmMSC series. (**E**) The circularity dynamics for mmSCs. (**F**) The circularity violin plot for all frames within the mmSC series. (**G**) The circularity dynamics for bmMSCs. (**H**) The circularity violin plot for all frames within the bmMSC series. The solid line shows the moving average (15 points for all curves). The black line inside the violin plot shows the mean value. (**A**,**B**,**E**,**F**) Abbreviations: 1–01; 2–02; 3-test01; 4-test02. (**C**,**D**,**G**,**H**) Abbreviations: 1-P8, 5K, 5%FBS; 2-P8, 5K, 10%FBS; 3-P8, 10K, 5%FBS; 4-P8, 10K, 10%FBS; 5-P10, 5K, 5%FBS; 6-P10, 5K, 10%FBS; 7-P10, 10K, 5%FBS; 8-P10, 10K, 10%FBS.

Time-lapse imaging allows for the tracking of the area and circularity of cells over time, enabling the researcher to not only identify average trends and population variability but also to assess heterogeneity and form variability, detect abnormal behavior, and quantitatively compare different experimental conditions. A striking example of this is the increase in area as confluence increases or the gradual decrease in circularity that occurs during the transition to the adhesive phenotype. According to a logistic model for bmMSCs, the average cell area per frame increases in time-lapse microscopy of cell cultures. When adhering to the substrate, cells flatten, reducing the circularity ratio as they elongate and thin their edges. This phenomenon is observed in the early phase after seeding: confluence increases due to spreading and flattening rather than division, resulting in an increase in the average area (Figure 3A) and a decrease in the average circularity.

The total area occupied by individual cells (Figure 3B) steadily increases regardless of the culture conditions, with the cells in 10% FBS growing faster than in 5% (Figure 3C,D), likely due to the richer supply of nutrients. Cell divisions appear as local fluctuations, wherein a part of the population suddenly decreases in size before resuming its growth. Higher seeding densities seem to result in slightly smaller cells overall, presumably due to more of them competing for the same nutrient supply.

The circularity of the cells begins to decline soon after plating (Figure 3E,F; first 50 frames), as they adhere to the bottom of the culture well plate and spread out, losing their circular shape in the process (Figure 3F). Compared with P10, cells at P8 show less pronounced changes in both of these parameters. Cells in experiments 5–8 (10% FBS and high passage) lost circularity faster and to a greater extent than cells at passage 8 (Figure 3H). This suggests that they committed earlier or more profoundly to an adherent, spread phenotype under these conditions. In contrast, the cells in media with a lower serum concentration remained slightly more rounded during the same period, consistent with a more gradual adhesion process.

The time series for circularity at P10 demonstrates the intensity of adhesive spread: a sharp decline in the early frames, followed by stabilization as focal adhesions form [21]. When approximated by a logistic function (e.g., 4P), the spread rate assessment demonstrates how, with equal technical error, intracellular factors influence the degree of cell stretching [22]. The time series of morphometric features (area, circularity) reflect not only the phenotypic responses to the environment but are also determined by genetic changes in cells [23,24]. For example, there is residual circularity with the construction of a multidimensional profile of cell phenotypes over time [25]. Graphs of area and circularity for each frame provide the means to visually evaluate the shape of the distribution (skewness, bimodality with mixed phenotypes). Comparing the distributions of two different conditions or time points (using non-parametric methods) reveals when the population begins to scatter in the form of measurable changes.

**Figure 4:**
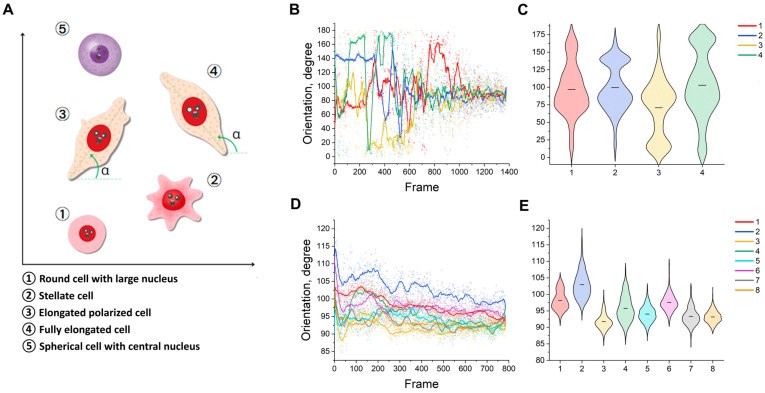
(**A**) Variants of the spatial configuration of cells; (**B**) graph of the angle of the semimajor axis to the abscissa axis by frame for mmSCs; (**C**) violin plot of mmSC orientation; (**D**) graph of the semimajor axis to the abscissa axis by frame for bmMSCs; (**E**) violin plot of bmMSC orientation. The solid line shows the moving average (15 points for all curves). The black line inside the violin plot shows the mean value. (**B**,**C**) Abbreviations: 1–01; 2–02; 3-test01; 4-test02. (**D**,**E**) Abbreviations: 1-P8, 5K, 5%FBS; 2-P8, 5K, 10%FBS; 3-P8, 10K, 5%FBS; 4-P8, 10K, 10%FBS; 5-P10, 5K, 5%FBS; 6-P10, 5K, 10%FBS; 7-P10, 10K, 5%FBS; 8-P10, 10K, 10%FBS.

We take the direction of the cell’s long axis as its orientation. In this case, the cell is described by a minimally bounding ellipse. Depending on its state at any given time, the cell can adopt an arbitrary spatial configuration. For example, it may appear as a round cell with a large nucleus. This is characterised by a compact body and a high nucleus-to-cytoplasm ratio, which is typical of small resting or progenitor-like cells. Alternatively, the cell may adopt a stellate form with an irregular outline and multiple protrusions, reflecting active spreading or motility. Alternatively, it may adopt an elongated, polarised shape with spindle-like morphology, a moderate nucleus-to-cytoplasm ratio, and an initial orientation along a defined axis. In an advanced state, the cell becomes fully elongated and shows a strongly polarised, spindle-like configuration, with the nucleus aligned to the long axis—a morphology that is characteristic of fibroblastic cells. Finally, the cell may present as a spherical form with a central nucleus, displaying rounded morphology and low adhesion or spreading. This may correspond to a non-adherent state, suspension or apoptosis. (Figure 4A), the angle is an arbitrary value from 0 to 180 degrees. Here, the values from 0 to 90 degrees and from 90 to 180 degrees are equivalent. The magnitude of the semimajor axis angle is based on a gradient of intracellular and intercellular attractants. The angle reflects the nature of intercellular interactions, which is particularly evident at low confluence values. At high confluence values, however, the average angle begins to vary within the 90 degree range (Figure 4B).

The cell orientation appears to be random, which is consistent with the parameters of the experiment (Figure 4E). It should be noted that, at the initial moment in time, the behavior of the MA curves (Figure 4D; first 50 frames) is consistent with that of the roundness, indicating the stage of adhesion to the surface. Cells cultured in 10% FBS seem to be overall more prone to orientation changes. We would like to draw the reader’s attention to the Friedman tests (our N_eff_ and permutation). It is based on the values at the point of intersection and appears to be statistically reliable (Supplementary File S1). The graph shows that the variance of the series decreases to values within the range of the curves. The most interesting values are those outside these curves and those identified by the Friedmann–Conover test. The proportion of outliers may indicate atypical objects. The angle values may indicate morphological heterogeneity caused by intracellular factors (Figure 4C).

**Figure 5:**
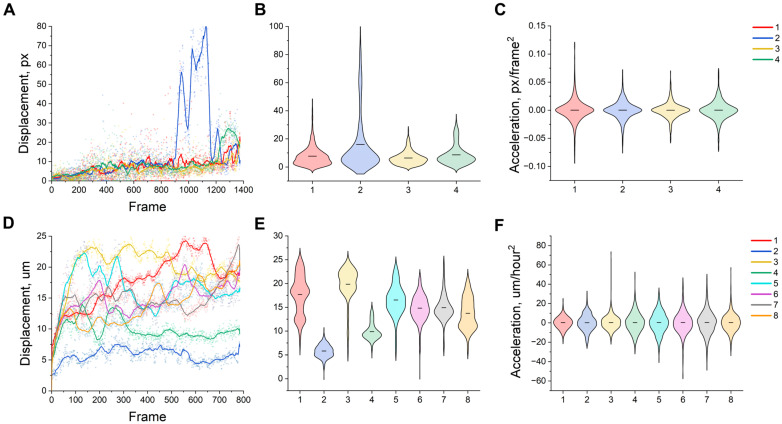
(**A**) Displacement over time for mmSCs, (**B**) displacement distribution for mmSCs by violin plot, (**C**) acceleration distribution for mmSCs by violin plot, (**D**) displacement over time for bmMSCs, (**E**) displacement distribution for bmMSCs by violin plot, (**F**) acceleration distribution for bmMSCs by violin plot. The solid line shows the moving average (15 points for all curves). The black line inside the violin plot shows the mean value. (**A**–**C**) Abbreviations: 1–01; 2–02; 3-test01; 4-test02. (**D**–**F**) Abbreviations: 1-P8, 5K, 5%FBS; 2-P8, 5K, 10%FBS; 3-P8, 10K, 5%FBS; 4-P8, 10K, 10%FBS; 5-P10, 5K, 5%FBS; 6-P10, 5K, 10%FBS; 7-P10, 10K, 5%FBS; 8-P10, 10K, 10%FBS.

As shown in Figure 5, some cells have distributions of displacement and acceleration that remain compact around the average value, while others show a significant right-sided shift in mass density. Thus, the curve for trajectory 2 exhibits distinct asymmetry: the majority of the density is distributed below the average value (black dotted line), yet the tail extends considerably to the right, indicating infrequent yet substantial displacement surges of up to approximately 80 px (Figure 5A,B). Similarly, the acceleration of the same trajectory (Figure 5C) has an extended upper quantile, indicating frequent peak accelerations. In contrast, trajectories 1 and 4 show almost symmetrical violin plots with a narrow range of values around the average, indicating a more stable creeping migration regime.

The relief distributions of displacement (Figure 5D,E) are unevenly scattered over the mass on the lower panel. Trajectories 1 and 6 are characterized by an upward shift in the main mass; their average displacement reaches 20–25 µm, and the upper quantile boundaries reach 30 µm, demonstrating active and unstable migration. In contrast, trajectory 2 remains ‘slow’: its density is concentrated below the average (~6 µm) with minimal tails. The acceleration distributions (Figure 5F) of trajectory 6 are noticeably expanded over all quartiles, indicating frequent and sharp changes in velocity. Other trajectories show narrower violin plots, corresponding to relatively smooth changes in kinetics. Cells cultured in 5% FBS display greater displacement, indicating greater motile activity. Notably, CD29 expression was found to be elevated in these cells, potentially suggesting a more active formation of connections to the culture plate required for the active cell movement. Furthermore, while cells at P10 tend to display greater changes in acceleration and deceleration, cells at P8 seem to be moving at more constant speeds.

We also observed a concurrent reduction in variability in some features exactly at these transition times. For instance, the frame-to-frame variance in orientation angle or circularity within each experiment temporarily dipped around the proliferation onset, perhaps because as cells commit to a new state, they become more behaviorally uniform for a period of time. In terms of statistics, it is intriguing that the Friedman test (Supplementary File S1) became significant precisely at the time these exponential curves diverged. In other words, the first detection of a statistically reliable difference between conditions coincided with the biological event of one group of cells exhausting their proliferative phase sooner. This reinforces that our smoothing and blocking approach is capturing real shifts in population behavior rather than random drift. Taken together, these results suggest that higher concentrations of serum in the culture media have a noticeable impact on cellular morphology and behavior. We quantitatively confirmed this divergence with a post hoc Conover and permutation test at the end of the time series, which showed that the final median circularity and final cell counts were significantly different between conditions.

To evaluate the extent to which the intrinsic lineage differences between adult stem cell subtypes are reflected in migratory and morphological phenotypes, we computed twelve complementary metrics covering displacement, directional bias, shape–motion coupling, and kinetic fluctuations. Although mmSCs and bmMSCs originate from distinct embryonic sources (the paraxial mesoderm and the somatic layer of the lateral plate mesoderm, respectively) and differ in differentiation potential and secretome, they are sometimes considered jointly as adult undifferentiated stromal cells to emphasize shared baseline features. The distinctions described below can be extended to this broader category with minimal modification. We bootstrapped each distribution (n = 10,000) and visualized the results side by side for the mmSC and bmMSC populations to determine whether developmental origin [26], secretome profile, or differentiation capacity correlates with changes in exploration range, velocity bursts, turning behavior, or arrest dynamics. Such comparative phenotyping reveals cell-type-specific motility signatures and provides empirical constraints for mechanistic models of cytoskeletal regulation and microenvironmental interaction.

**Figure 6:**
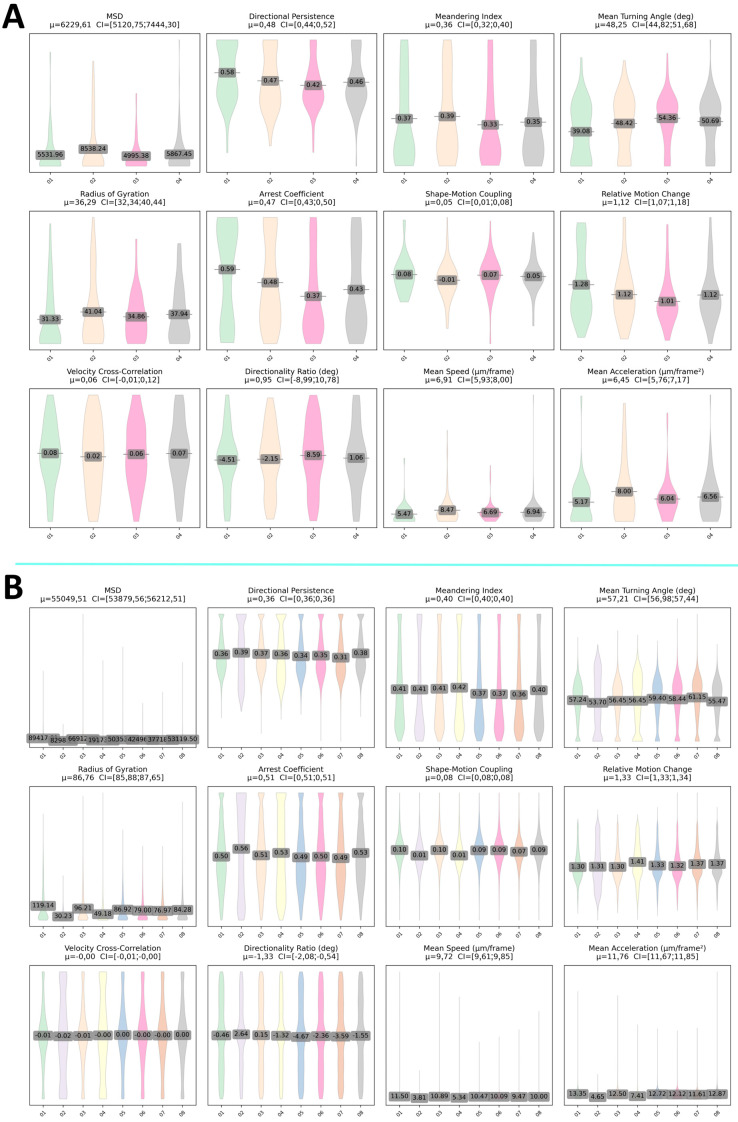
Violin plots of bootstrapped distributions (n = 10,000) for twelve morphodynamic and kinetic metrics in two stem cell populations: (**A**) mmSCs Abbreviations: 1–01; 2–02; 3-test01; 4-test02 (**B**) bmMSCs Abbreviations: 1-P8, 5K, 5%FBS; 2-P8, 5K, 10%FBS; 3-P8, 10K, 5%FBS; 4-P8, 10K, 10%FBS; 5-P10, 5K, 5%FBS; 6-P10, 5K, 10%FBS; 7-P10, 10K, 5%FBS; 8-P10, 10K, 10%FBS). In each subplot, the black horizontal bar indicates the mean, and the overlaid label provides the 95% bootstrap confidence interval. The metrics are shown in the following order: (1) mean squared displacement (MSD), (2) directional persistence, (3) meandering index, (4) mean turning angle (°), (5) radius of gyration, (6) arrest coefficient, (7) shape–motion coupling, (8) relative motion change, (9) velocity cross-correlation, (10) directionality ratio (°), (11) mean speed (µm/frame), (12) mean acceleration (µm/frame^2^).

In the mmSC population (Figure 6A), metrics of spatial exploration and movement magnitude exhibit a pronounced right skew. The MSD (mean ≈ 6.2 × 10^3^ px^2^, 95% CI [5.1 × 10^3^, 7.4 × 10^3^]) and the radius of gyration (mean ≈ 36.3 px, 95% CI [32.3, 40.4]) suggest that the majority of cells remain in close proximity to their origin, while a minority undertake significantly more extensive excursions. Similarly skewed metrics include mean speed (mean ≈ 6.9 µm/frame, 95% CI [5.9, 8.0]) and mean acceleration (mean ≈ 6.5 µm/frame^2^, 95% CI [5.8, 7.2]), which are consistent with episodic bursts of rapid movement superimposed on a slower baseline. The directionality ratio remains centered near zero, reflecting no persistent systematic drift and suggesting that the net displacement efficiency relative to path tortuosity is neutral. Shape–motion coupling is low and variable, and the arrest coefficient (approximately 0.47) indicates moderate dwell times in low-motility states.

In the bmMSC population (Figure 6B), the same metrics shift to substantially larger values while retaining their asymmetric profiles. The mean squared displacement (MSD) increases by nearly an order of magnitude (5.5 × 10^4^ px^2^, [5.4 × 10^4^, 5.6 × 10^4^]), and the radius of gyration roughly doubles (86.8 px, [85.9, 87.7]), indicating more extensive spatial roaming. Mean speed (9.7 µm/frame, [9.6, 9.8]) and acceleration (11.8 µm/frame^2^, [11.7, 11.9]) are elevated and right-skewed, consistent with frequent high-velocity and high-acceleration events. Despite this enhanced locomotor vigor, the directionality ratio remains close to zero (approximately −1.3°), showing that the overall displacement efficiency is preserved. bmMSCs display slightly more consistent shape–motion coupling and a modestly higher arrest coefficient (approximately 0.51), along with increased fluctuations in relative motion, which may reflect more distinct ‘start–stop’ kinetics in their migration.

Intrinsic, lineage-associated differences do not manifest in baseline directional bias, which is comparably neutral in both mmSC and bmMSC populations, but rather in the scale and temporal structuring of motility. bmMSCs exhibit broader and more energetic exploratory behavior, with an elevated mean squared displacement (MSD), radius of gyration, speed, and acceleration, as well as more pronounced kinetic fluctuations. This suggests a more dynamic cycle of active translocation and pausing. The relatively stable yet subtle differences in shape–motion coupling and arrest dynamics indicate the distinct integration of morphology and movement programs across these lineages. These phenotypic signatures provide quantitative constraints for mechanistic models of cytoskeletal regulation and microenvironmental interaction that vary according to developmental origin.

## 3. Materials and Methods

### 3.1. Bone Marrow MSC Isolation and Culture

To obtain murine MSCs, the bone marrow was removed from the bones of the eight Black 6 (C57Bl/6) mice and washed with 5 mL of PBS into a sterile Petri dish using a 22-gauge needle. Bone marrow cells were layered on top of an equal volume of ice-cold Ficoll–Paque (1.077 g/mL) solution in a 15 mL sterile conical tube and centrifuged at 1900 rpm for 20 min at 4 °C without braking. The mononuclear cell-containing interphase was transferred to a new 15 mL conical tube and washed with 10 mL of cold sterile PBS, followed by centrifugation at 1400 rpm for 15 min at 4 °C. The supernatant was discarded, and the washing step was repeated. The cell pellet was resuspended in prewarmed DMEM-High Glucose (PanEco, Moscow, Russia) supplemented with 20% FBS (neoFroxx, Einhausen, Germany) and 1% GlutaMax (Gibco, Waltham, MA, USA) and plated onto 6-well plates with a seeding density of 15 million cells per well. The cells were allowed to adhere for 5 days, after which the media was changed. The cells were then cultured for a week, changing the media every 2–3 days. After the first week the media was exchanged for DMEM-F12 (PanEco, Russia) supplemented with 10% FBS (neoFroxx, Germany) and 1% GlutaMax (Gibco, USA) and was likewise changed every 2–3 days. Upon reaching ~90% confluency, the cells were passaged with 0.1% trypsin (Servicebio, Wuhan, China), and a subset of cells was plated in 48-well plates (NEST, Wuxi, China) for imaging (at 5k and 10k per well) in media supplemented with either 5% or 10% FBS for both cell densities (Figure 1). Passage 8 and 10 cells were used in the experiment.

### 3.2. RNA Extraction and qRT-PCR

For quantitative real-time PCR (qRT-PCR) analysis, MSCs were cultured in a 6-well plate in media containing 5% or 10% FBS until reaching confluency, after which they were lysed using the phenol guanidine thiocyanate solution “Lira Carib” (Biolabmix, Novosibirsk, Russia). RNA was extracted from the lysates according to the manufacturer’s instructions. RNA concentration and purity were assessed with the NanoDrop OneC spectrophotometer (Thermo Fisher Scientific, Waltham, MA, USA); samples with absorbance ratios of A260/A280 ≥ 1.9 and A260/A230 ≥ 2.0 were selected for analysis. cDNA synthesis was carried out using the Reverse Transcriptase RNAscribe RT kit (Biolabmix, Russia) according to the manufacturer’s instructions, using 5 ug of total RNA for each sample. qPCR reactions were set up using 500 ng of cDNA per reaction in BioMaster HS-qPCR SYBR Blue (2×) master mix (Biolabmix, Russia) according to the manufacturer’s instructions and forward (F) and reverse (R) primer sets (Table 1).

Three biological replicates, each consisting of four technical replicates, were analyzed. *GAPDH* was used as the housekeeping gene control. Amplification reactions were performed using a StepOne Plus thermal cycler (Applied Biosystems, Knutsford, UK). Samples cultured in 10% FBS (the more commonly used concentration) were used as controls. Relative gene expression was determined using the 2^−ΔΔCt^ method. The normal distribution of the data for each group was verified using the Shapiro–Wilk test. An unpaired *t*-test was used to compare gene expression between groups, and the results were considered statistically significant at *p* < 0.05. All statistical tests were performed using GraphPad Prism version 8.2.1 (GraphPad Software, Boston, MA, USA).

### 3.3. Time-Lapse Data and Cell Segmentation

The implementation utilizes frameworks such as PyTorch 2.6.0 to ensure high flexibility and computational efficiency on GPUs. In the experimental set up, the data are normalized, converted into tensor format, and divided into training and validation sets, with special care taken to ensure reproducibility by setting random number generators and limiting the number of threads. All computational analyses, including model training and data processing, were performed using NVIDIA A100 GPUs (NVIDIA Corporation, Santa Clara, CA, USA), which provided the necessary computational power for handling large datasets and training deep learning models efficiently.

We curated a diverse collection of annotated brightfield images spanning multiple human and murine cell types, including peripheral blood mononuclear cells, bronchial epithelial cells, skeletal myoblasts, dental pulp progenitors, dystrophin-deficient myoblasts, adipose-derived stem cells, oxidatively stressed stem cells, and late-passage mesenchymal stem cells. All annotations follow the YOLO format and are publicly available via Zenodo.

**Figure 7:**
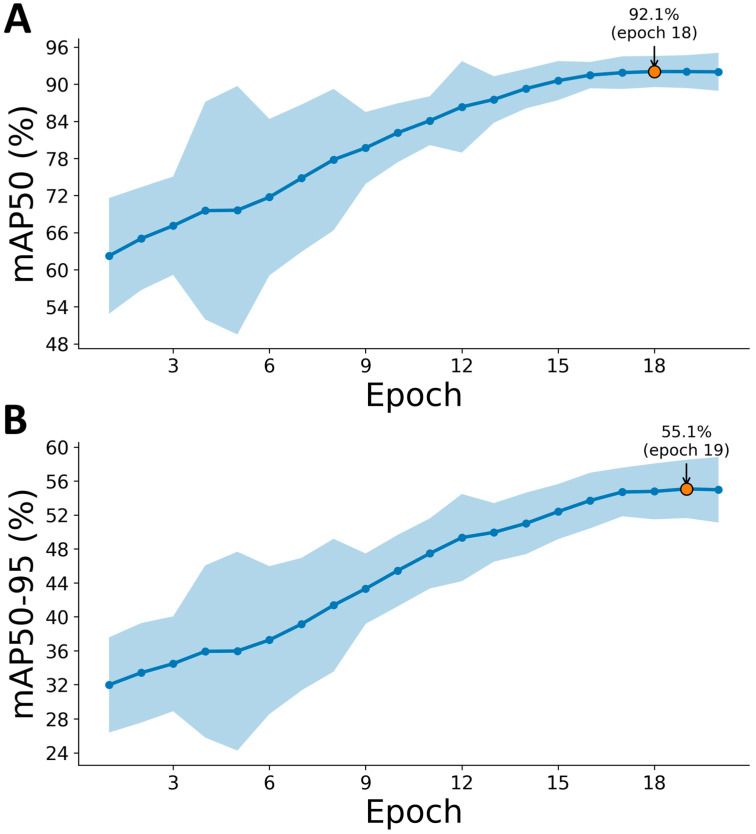
Training dynamics of object detection performance. (**A**) Mean average precision at IoU = 0.50 (mAP50). (**B**) Mean average precision averaged across IoU thresholds 0.50–0.95 (mAP50-95).

For the dynamic studies, live cell videos of mesenchymal stem cells were acquired under different culture conditions using an AxioObserver 7 motorized microscope (Carl Zeiss Microscopy GmbH, Jena, Germany) with a heating insert (Okolab, Rovereto, Italy). Frames were captured at fixed 5 min, 30 s intervals, resulting in approximately 800 frames per movie. Cell detection and segmentation employed a two-stage deep learning pipeline: first, a YOLO v11 (Figure 7), fine-tuned on our annotated images, localized each cell with bounding boxes; then, a lightweight segmentation head converted these proposals into precise binary masks.

The segmentation performance was rigorously assessed against expert-curated masks, achieving mean mAP50 scores of approximately 0.92 and mAP50-95 values of around 0.55. All subsequent morphometric and motility analyses were performed using these validated masks to ensure consistent feature extraction across experimental conditions.

### 3.4. Time-Stamped and Time-Dependent Feature Analysis

The raw frames were first corrected for uneven illumination using flatfield normalization, and then de-noised using a non-local means filter. All subsequent analyses were performed using the pre-processed grayscale images with a calibrated pixel size of 0.5199 µm. Cell outlines were delineated in each frame using a combination of adaptive thresholding and morphological filtering to generate binary masks. Small artifacts were removed by area filtering (<100 px^2^), and concave regions were smoothed via morphological closing. Unique labels were assigned to individual masks, and centroids were computed as the mean of all boundary pixels. To reconstruct cell trajectories, the centroids were linked across frames via nearest-neighbor matching, subject to a maximum displacement constraint of 10 µm per time step. Gaps of up to two frames were bridged via linear interpolation.

All analyses were implemented in Python 3.9, with the following libraries used for specific tasks: OpenCV for image operations, Shapely for geometric calculations, NumPy and Pandas for data handling, and SciPy (with the optional scikit-posthocs package) for statistics. Multi-threading was employed to accelerate feature computation across thousands of cell masks. The complete processing pipeline and example datasets are available in the public A/B Cell Policy repository under an open-source license. All graphical plots were performed using OriginPro version 2025b (OriginLab Corporation, Northampton, MA, USA).

### 3.5. Comprehensive Quantitative Profiling of Cell Migration Dynamics

In static morphometric analysis, four intuitive shape descriptors are extracted from each cell outline in brightfield images [27]. First, we measure the cell’s two-dimensional footprint, or projected area, which reflects cell size and can signal growth, spreading, or contraction. Next, we quantify the cell’s deviation from a perfect circle, or elongation, by comparing the longest and shortest axes of an equivalent ellipse [28]. This property is often linked to polarization or migratory readiness [29]. We also record the main orientation of the ellipse to give the tilt of the cell’s long axis relative to the imaging frame. This can reveal collective alignment or directional cues. Finally, we compute a smoothness index of the cell boundary, indicating whether the cell margin is regular and compact or irregular and protrusive. These features hint at membrane dynamics, protrusion formation, and adhesion patterns.

Together, these four descriptors provide a concise yet powerful geometric fingerprint of each cell in brightfield microscopy. As brightfield images lack fluorescent labels, cell morphology is often the richest source of phenotypic information. Size changes can indicate the cell cycle and spreading, elongation and orientation can reveal polarity and alignment, and irregularities in the boundary can reflect active protrusions or blebbing. Integrating these shape metrics thus enables the sensitive detection of subtle morphological shifts, the quality control of segmentation, and the comparison of phenotypes across conditions, even when contrast and signal are inherently limited [30].

In order to capture the full complexity of cell motility, we computed a wide range of dynamic descriptors for each reconstructed trajectory [31,32]. While some of these metrics may appear redundant at first, collectively they analyze distinct aspects of movement, such as statistical, geometric, temporal, and shape–motion coupling [33]. This allows for the sensitive detection of subtle phenotypic differences, condition-specific behaviors, and mechanistic inferences. The rationale behind each calculated parameter is detailed below.

MSD quantifies how far a cell wanders, on average, from its point of origin over time [34,35]. By calculating the average of the squared distances from the initial location, MSD reveals whether the motion is sub-diffusive (confined), diffusive (random walk), or super-diffusive (directed). Differences in MSD scaling exponents may indicate changes in the generation of underlying forces, the dynamics of cytoskeletal remodeling, or extracellular interactions.

Defined as the mean cosine of consecutive turning angles, directional persistence measures the tendency of a cell to maintain its instantaneous direction of motion [36]. Values near +1 denote straighter, more persistent runs, whereas values near 0 or negative indicate random or frequently reversing paths. This metric is critical for distinguishing, for example, chemotactic bias (high persistence) from purely exploratory motility.

The meandering index, also known as path straightness, is the ratio of net displacement (the straight line distance between the start and end points) to total path length. Unlike persistence, which focuses on local angular changes, the meandering index provides a global assessment of how much a cell’s travel contributes to net progress [37]. This helps to distinguish between cells that meander extensively and those that make efficient translocations.

The mean turning angle was computed as the mean absolute angular deviation between successive velocity vectors, expressed in degrees. By averaging these angles, this descriptor captures the typical sharpness of a cell’s directional changes at each time step, reflecting its interactions with the microenvironment or intrinsic steering behaviors [38,39].

The radius of gyration is defined as the root mean square distance of all centroid positions from the center of mass of the trajectory [40]. This metric quantifies the cell’s spatial spread over the entire tracking period, with larger values indicating broader coverage and the potential for increased invasive or search-like activity.

The arrest coefficient was calculated as the proportion of time frames in which the instantaneous speed fell below a specified threshold (0.2 µm/frame for ours). By identifying intervals of almost-zero movement, this parameter highlights periods of pause or adhesion-mediated arrest that alternate with active migration phases [41].

The shape–motion coupling was assessed using the Pearson correlation coefficient, which was calculated for each frame based on cell area and instantaneous speed. A strong positive correlation indicates that morphological spreading drives faster translocation, whereas a weak or negative correlation suggests the more independent regulation of shape and motility [42].

Relative motion change was defined as the mean absolute difference in speed between consecutive frames, normalized by the overall mean speed. This dimensionless index quantifies abrupt accelerations or decelerations, capturing the irregularity of a cell’s motile behavior over time.

The velocity cross-correlation was calculated as the Pearson correlation coefficient of the x- and y-components of the interframe displacement vectors. Positive values indicate coordinated movement along diagonal trajectories, while values close to zero or negative reflect isotropic or orthogonally biased motion patterns.

The directionality ratio was computed as the ratio of the net displacement (the straight line distance from the start to the endpoint) to the total path length. This index evaluates the efficiency of overall translocation, with higher values indicating that a greater proportion of the traveled distance contributes to net progress.

Frame-to-frame displacement was computed as the Euclidean distance between a cell’s centroid in the current frame and its position in the immediately preceding frame [43]. All distances were converted to micrometers. This per-frame displacement is the fundamental measure of cell movement over successive time points, underpinning subsequent derivations of speed and acceleration.

Instantaneous speed was calculated by dividing each frame-to-frame displacement by the interframe interval (350 s in the present experiments). Expressed in micrometers per frame, this descriptor quantifies the cell’s translational rate at each time point, thereby enabling the identification of rapid motile bursts versus slower crawling behavior. Instantaneous acceleration was defined as the change in instantaneous speed between consecutive frames, normalized by the same interval [44]. By measuring the rate of velocity change, this parameter captures transient accelerations and decelerations arising from force generation and cytoskeletal remodeling.

Displacement, speed, and acceleration all feature in both the per-frame (static) summaries and the track-level (dynamic) analyses because they fulfill dual roles. For dynamic profiling, instantaneous values are first computed at each time point for an individual cell, and then averaged over the entire lifespan of its trajectory [45]. This characterizes the kinetic signature of that cell. In contrast, static metrics are obtained by averaging instantaneous values across all cells present at each frame, providing a cross-sectional snapshot of population-level motility. Consequently, dynamic aggregates reveal the temporal evolution of individual trajectories, whereas static aggregates reflect the collective behavior of the cell ensemble at specific time points.

### 3.6. Integrated Non-Parametric Analysis: Bartlett-Corrected and Permutation Friedman Tests for Autocorrelated Cell Tracking Time Series

We demonstrate that the delay in shooting in sequential scan mode means that experiments in individual scenes and fields of view cannot be compared directly. We solved this problem by using the Bartlett approximation when adapting Friedman’s degrees of freedom rather than using the Friedman permutation test. The raw time series of cell metrics exhibited short-term fluctuations, so we first applied a fixed window length (N ~15 frames) moving average (ma) filter to reduce high-frequency noise and markedly decrease the variance of the original series. Let X_ma_(t) denote the smoothed series. As adjacent time series frames are autocorrelated, we estimated the lag-1 autocorrelation (Q) in each window. We then calculated the effective sample size N_eff_ using Bartlett’s formula (Equation (1)) for an approximate AR (1) process [46] as follows:

(1) $$N_{eff}=N\;\times \frac{\left(1-Q\right)}{\left(1+Q\right)}$$

(2) $$Q=\frac{\sum_{t=1}^{N-1}\left(Xₘₐ(t)-\overset{-}{X})\cdot (Xₘₐ(t+1)-\overset{-}{X}\right)}{\sum_{t=1}^{N}(Xₘₐ(t)-\overset{-}{X}{)}^{2}}$$

Substituting N_eff_ for N corrects the degrees of freedom for temporal autocorrelation in subsequent statistical tests. To compare the different culture conditions, we treated each frame position within the sliding window as a block and each experimental series as a treatment. The observations within each block were rank-transformed, and the Friedman Q statistic (Equation (2)) was computed using Bartlett-corrected degrees of freedom N_eff_. This preserves the non-parametric inference of Friedman’s test while adjusting for autocorrelation. This approach enables us to control the distribution of ma values resulting from the properties of sequential scanning (boustrophedon scanning) used during data acquisition (ma on Supplementary Files S1 and S2 for each plot).

To guard against any violation of the asymptotic χ2 approximation, particularly in windows with strong autocorrelation or unusual rank distributions, we implemented a permutation-based Friedman test (RAW on Supplementary File S1 for each plot). For each sliding window, the treatment labels were randomly permuted within blocks 1000 times to generate an empirical null distribution of Friedman’s statistics. The permutation *p*-value was then calculated as the proportion of these permuted statistics that exceeded the observed statistic. This provides an exact significance test under minimal assumptions.

When the Friedman approach indicated overall significance (*p* < α = 0.05), we performed Conover’s post hoc comparisons with Holm’s correction to control the family-wise error rate. Conover’s method operates on within-block ranks, providing robust pairwise rank-sum tests that are unaffected by non-normality and unequal variances. Our pipeline reduces noise, accounts for temporal autocorrelation, and rigorously assesses treatment effects without assuming normality or equal variances by combining MA filtering with both Bartlett-corrected and permutation-based Friedman tests. Heatmaps of significant pairwise comparisons highlight intervals where the majority of treatment pairs reject the null hypothesis, and permutation *p*-values corroborate these findings. This integrated non-parametric framework ensures the robust detection of dynamic differences in cell tracking metrics.

## 4. Conclusions

The repository is a fully functional toolkit for the automated analysis of time-lapse images of cell cultures, consisting of two complementary modules. The first, A/B CellTracker, uses advanced segmentation algorithms to implement a reliable skeleton for tracking cells in consecutive frames, calculating shape and intensity features, and performing hybrid matching by IoU, distance, feature, and geometric metrics. It also has the ability to reconsolidate identity during separation/fusion. The second module, A/B CellAnalyser, performs post-tracking analysis of the obtained data. It extracts static morphometric parameters (area, eccentricity, roundness, and orientation); calculates dynamic migration (MSD, meandering index, directional preservation, speed, and acceleration); aggregates results by frames and tracks; conducts statistical comparisons (Friedman’s test and post hoc analyses); and stores all conclusions in visual tables and graphs for subsequent biological interpretation. We provide labeled data for scaling up, paying special attention to areas such as time-lapse photography, which will play an essential role in shaping the multimodal cellular world.

We gained an in-depth understanding of the behavior of bmMSCs under different conditions by combining classical morphometric analysis with time series statistics, automated tracking, and advanced modeling. Descriptive statistics revealed baseline similarities and differences in morphology and growth (e.g., a faster loss of circularity and an earlier area plateau in treated cells). Time series analysis using moving averages and non-parametric tests identified the precise timing of divergence between experiments, confirming an accelerated transition in the perturbed condition.

When we compare the available tools, our model occupies a distinctive niche. While commercial platforms such as Imaris version 10.2.0, Arivis Vision4D version 4.2 and Amira version 2024.2 are powerful, they are licence-dependent and GUI-oriented. Free frameworks such as Ilastik version 1.4.1, DeepCell version 0.12.10 and TrackMate version 7.1.0 provide either segmentation or basic trajectory analysis, but not both. By contrast, A/B Cell Policy integrates morphological descriptors and advanced dynamic indicators within a single open-source pipeline. It automates the entire segmentation, tracking, morphometry, statistical testing, and visualization cycle, eliminating the need to transfer data between different packages. Furthermore, it incorporates robust statistical engines (e.g., Friedman’s tests adjusted for effective sample size, permutation checks, and bootstrap confidence intervals), multithreaded execution, and customizable parameters, while ensuring reproducibility and transparency. This makes the toolkit a practical solution for immediate biological interpretation and a launchpad for predictive models based on successor features, diffusion transformers, or world models.

By explicitly modeling the policies and rewards underlying these outcomes, our aim is to achieve a mechanistic understanding of cellular decision-making. This knowledge will be invaluable for guiding interventions in regenerative medicine and stem cell therapy, where it may be necessary to steer cells towards or away from specific fates. Finally, our data highlight that even relatively minor variations in culture conditions can significantly impact MSC properties, emphasizing the importance of standardized protocols and transparent reporting. Such an approach creates in vitro disease models for which the use of stem cells and their unique ability to differentiate is the only way to model pathological processes and evaluate the efficiency of various therapeutic approaches.

## Figures and Tables

**Table 1:** Primer sequences for MSC marker genes, shown in 5′-3′ direction.

Gene Name	F Primer	R Primer
CD29	GGACGCTTACTGCAGGAAAGAG	ACAGTCACAKGCRCTGCCAGTG
CD34	GGAGCCACCAGAGCTAYTCC	CCTGGCCTCCACCRTTCTCC
CD44	CCTCGTCACGTCCAACACCTCC	TCGATGGTGGAGCCGCTGC
CD73	CCTGTGGTCCAGGCCTATG	GCTTTGATGGTCGCATCTTCAG
CD105	CGCTTCAGCTTCCTCCTCCG	CACCACGGGCTCCCGCTTG

## Data Availability

The data presented in this study are openly available in [ABCellPolicyat [https://doi.org/10.5281/zenodo.16480754 (version 0.1)], reference number [https://github.com/ElijahBiocinth/ABCellPolicy] (accessed on 28 August 2025), under a Creative Commons Attribution 4.0 International license.

## Supplementary Materials

The following supporting information can be downloaded at: https://www.mdpi.com/article/10.3390/ijms26178455/s1.

## Abbreviations

The following abbreviations are used in this manuscript:

MSC	Mesenchymal stem cell
YOLO	You Only Look Once
SOTA	State of the Art
IoU	Intersection over Union
FBS	Fetal bovine serum
bmMSCs	Mouse bone marrow MSCs
ISCT	International Society for Cellular Therapy
mmSC	Mouse muscle SC
MA	Mean average
CI	Confidential interval
MSD	Mean square displacement
