# Supplementary material for: Deriving the A/B Cells Policy as a Robust Multi-Object Cell Pipeline for Time-Lapse Microscopy
Source: Int J Mol Sci. 2025 Aug 30;26(17):8455. doi: 10.3390/ijms26178455 (PMC12429040; doi:10.3390/ijms26178455)

Heat maps illustrate the dynamics of statistically significant differences between conditions, as determined by the non-parametric Friedman test, within a block structure (scenes/replicas). Time (frames) is shown horizontally and pairs of metrics are shown vertically (see Methods). The colour encodes the number of significant comparisons at the corresponding point in time after multiple time checks (FDR Benjamini–Yekutieli), ranging from deep purple/red (no differences) to light (the largest number of differences). Two versions are shown for each metric: RAW (Friedman permutation) and MA (Friedman with Bartlett correction). Continuous layers of saturated colour reflect extended intervals of divergence between conditions, while rare isolated cells correspond to transients. Collectively, the maps demonstrate that the RAW method reveals both transient and long-term discrepancies, the MA method confirms stable effects and eliminates short-term noise, and the most stable divergence can be observed in orientation and eccentricity. In contrast, area and roundness more frequently reflect brief phases of morphological restructuring. These significance windows identify candidates for critical cell states and define time intervals for mechanistic analysis.

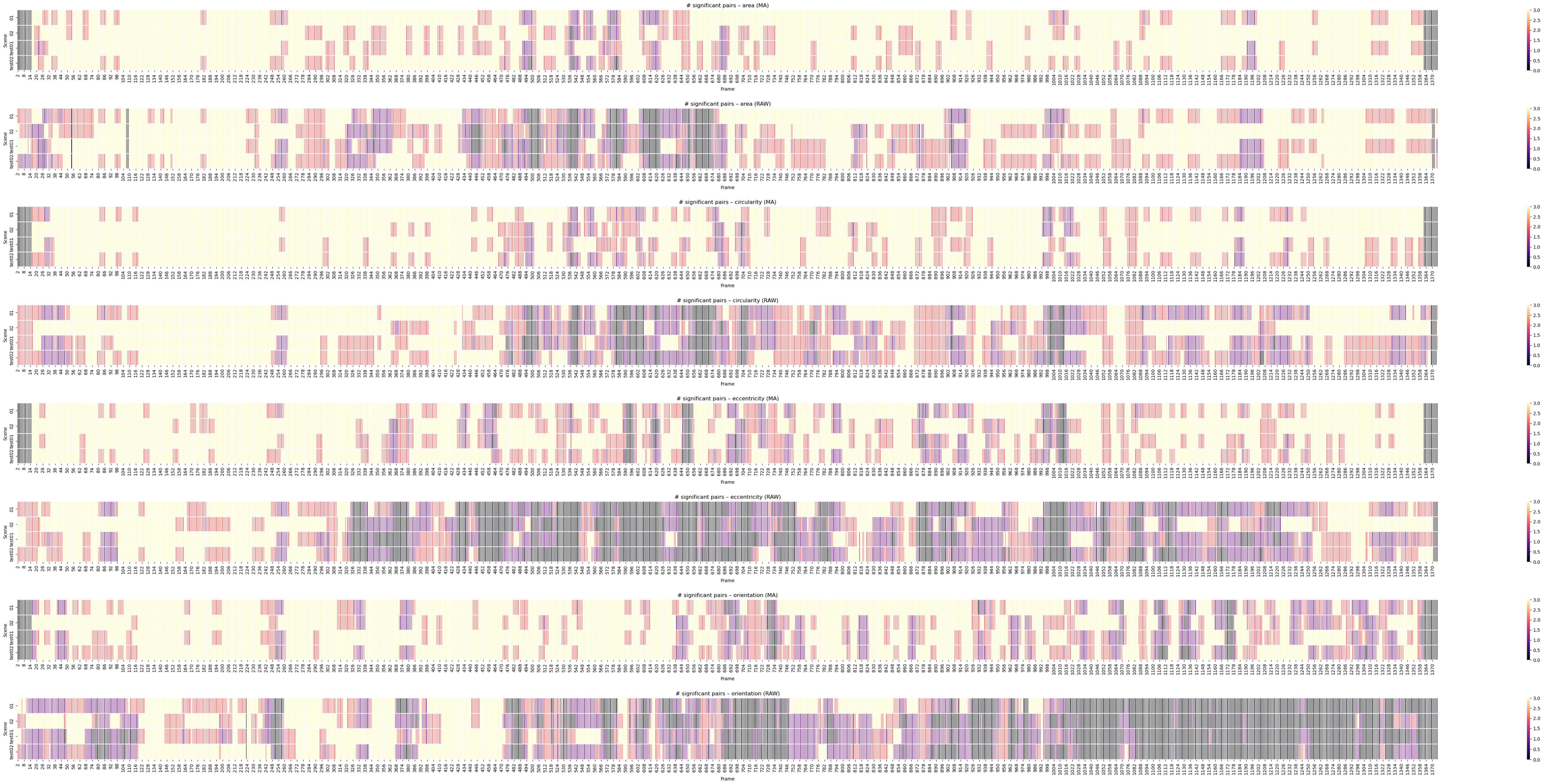

Supplement: Supplementary file 1 [file ijms-26-08455-s001.zip › ijms-3826320-supplementary s1.pdf]
